# Changes in novel haematological parameters following thermal injury: A prospective observational cohort study

**DOI:** 10.1038/s41598-017-03222-w

**Published:** 2017-06-12

**Authors:** R. J. Dinsdale, A. Devi, P. Hampson, C. M. Wearn, A. L. Bamford, J. Hazeldine, J. Bishop, S. Ahmed, C. Watson, J. M. Lord, N. Moiemen, P. Harrison

**Affiliations:** 1 The Scar Free Foundation Birmingham Centre for Burns Research, Birmingham, UK; 2 Institute of Inflammation and Ageing, University of Birmingham, Birmingham, UK; 3 Department of Haematology, Queen Elizabeth Hospital, Birmingham, UK; 4 NIHR Surgical Reconstruction and Microbiology Research Centre, University Hospitals Birmingham NHS Foundation Trust, Birmingham, UK; 5 Queen Elizabeth Hospital Birmingham, University Hospitals Birmingham NHS Foundation Trust, Birmingham, UK

## Abstract

The mortality caused by sepsis is high following thermal injury. Diagnosis is difficult due to the ongoing systemic inflammatory response. Previous studies suggest that cellular parameters may show promise as diagnostic markers of sepsis. The aim of this study was to evaluate the effect of thermal injury on novel haematological parameters and to study their association with clinical outcomes. Haematological analysis was performed using a Sysmex XN-1000 analyser on blood samples acquired on the day of the thermal injury to 12 months post-injury in 39 patients (15–95% TBSA). Platelet counts had a nadir at day 3 followed by a rebound thrombocytosis at day 21, with nadir values significantly lower in septic patients. Measurements of extended neutrophil parameters (NEUT-Y and NEUT-RI) demonstrated that septic patients had significantly higher levels of neutrophil nucleic acid content. A combination of platelet impedance count (PLT-I) and NEUT-Y at day 3 post-injury exhibited good discriminatory power for the identifying septic patients (AUROC = 0.915, 95% CI [0.827, 1.000]). Importantly, the model had improved performance when adjusted for mortality with an AUROC of 0.974 (0.931, 1.000). A combination of PLT-I and NEUT-Y show potential for the early diagnosis of sepsis post-burn injury. Importantly, these tests can be performed rapidly and require a small volume of whole blood highlighting their potential utility in clinical practice.

## Introduction

Thermal injuries are a common and debilitating form of traumatic injury with approximately 6 million people each year globally receiving medical care^1^. Thermal injury results in profound changes in a number of metabolic, haematological and inflammatory responses which may lead to poor outcome post thermal injury^2^. Although advancements in burn care have improved immediate patient outcomes^3^, the prevalence of sepsis and its associated mortality remains significant^4^. Of note, the diagnosis of sepsis represents a major clinical challenge, as many of the classically used diagnosis criteria are masked by the ongoing systemic inflammatory response syndrome (SIRS)^5^.

Following traumatic injury, circulating biomarkers of sepsis have been identified. However, although exhibiting both prognostic and diagnostic potential many of these biomarkers, as single entities, lack sensitivity and specificity for early diagnosis of sepsis^6–9^. In an attempt to overcome such issues recent studies have investigated whether a combination of biomarkers offer greater diagnostic potential^6, 10^. For example, in a recent study of severely injured trauma patients, a combination of patient immune status coupled with measurement of interleukin-6 concentrations improved both the specificity and positive predictive value compared to the cytokine data alone^10^. Also, we have recently described in a cohort of patients with thermal injury that a combination of clinical and laboratory markers (Immature granulocytes (IGs), neutrophil function and revised Baux Score obtained at day 1 post-injury), demonstrates good discriminatory power to predict the later development of sepsis (AUROC 0.986)^6^.

Here, in this study we aimed to expand upon our recent publication by providing a more detailed analysis on whether early changes in a variety of novel cellular parameters also have potential clinical utility in a cohort of patients with thermal injury. The Sysmex XN-1000 is a high throughput state of the art haematology analyser that provides a rapid full blood count with a number of extended novel parameters of potential interest in an intensive care unit setting. Novel extended parameters include a simultaneous measurement of IGs, Fragmented Red Cells (FRC) and reticulocytes, and a new accurate platelet count (PLT-F) coupled with the immature platelet fraction (IPF)^11^.

Our preliminary data in thermally injured patients suggest that there are early significant changes in the bone marrow production of neutrophils resulting in early appearance of the IG population^6^. Although quantification of this subset of cells certainly has potential diagnostic application for sepsis, the Sysmex XN also reports five other neutrophil parameters that study the properties of the entire neutrophil population and IGs in more detail^6, 12^. These include neutrophil granularity index (NEUT-GI), neutrophil reactive intensity (NEUT-RI), measurement of cellular nucleic acid content (NEUT-Y), measurement of inner granularity (NEUT-X) and a vector sum of NEUT-X and NEUT-Y (NEUT-Z).

Additionally, there is evidence of an increased platelet turnover in patients with severe burns that results from a fall in platelet count with a nadir typically at day 3 followed by an increase in platelet production and a rebound thrombocytosis peaking at around day 1 post-injury^9^. In a study of 244 patients, Marck *et al*. reported that lower platelet counts at days 3 and 15 are associated with poor patient outcomes suggesting that abnormal bone marrow responses are important in thermal injury^9^. Interestingly, this study was performed using impedance counting technology, which is prone to interference, potentially leading to overestimation of platelet counts in samples where FRC are present in high concentrations^13^.

Thermal injury causes direct damage to circulating red blood cells that can result in red cell fragmentation with the appearance of large numbers of microspherocytes causing overestimation of platelet counts by impedance analysers^13, 14^. The Sysmex XN-1000 offers a solution to this problem by not only measuring 3 platelet counts in parallel (namely impedance (PLT-I), optical (PLT-O) and the more accurate fluorescent count (PLT-F)), a measure of platelet production (IPF) but also providing quantification of FRC. Thus, we can therefore study the effect of thermal injury on the generation of FRC, platelet counts and whether accurate platelet counts and platelet production are still useful in predicting sepsis and poor outcomes post-burn.

In this observational study we aimed to evaluate the effect of thermal injury on neutrophil, FRC and platelet kinetics using the Sysmex XN-1000 analyser. Data from 39 patients was collected longitudinally following thermal injury and compared to data from 40 healthy volunteers (control cohort). In addition, the predictive capacity of each cellular parameter either alone or in combination was evaluated for the early prediction of clinical outcomes, with particular focus on sepsis.

## Results

### Patient demographics

A total of 39 adults and 40 healthy adult volunteers (control cohort) were included in the study. The mean age of patients with thermal injuries was 46 years (range 16–88) and the mean burn size was 38% TBSA (range 15–95). The incidence of sepsis in this cohort was 69%, with 27 patients experiencing one or more episodes during their hospital stay. The median time to first septic episode in this cohort was 5 days post injury (interquartile range 4–9). The median time to first episode of multiple organ failure in this cohort was 4 days post injury (interquartile range 2.5–9.5). Detailed patient demographics are displayed in Table 1.

**Table 1:** Patient demographics.

Characteristic	All (n = 39)	Sepsis (n = 27)	No Sepsis (n = 12)	P (sepsis vs non sepsis)
Age, y	46	45	46	ns
Gender (M:F)	27:12	19:8	8:4	ns
ABSI	8.4	9.3	6.6	0.0018
%TBSA	38	45.3	21.8	0.0003
%FT burn	28	34.6	14.0	0.0029
Survived (Y:N)	26:13	15:12	11:1	0.0272
Inhalation injury (Y:N)	20:19	17:10	3:9	0.0286

Sepsis and no-sepsis variables were analysed by Mann-Whitney (continuous variables) or Chi-squared test (categorical variables). Abbreviations: ABSI = abbreviated burn severity index, %TBSA = percentage total body surface area, %FT = percentage full thickness burn.

### Thermal injury results in a classical platelet kinetic profile

**Figure 1:**
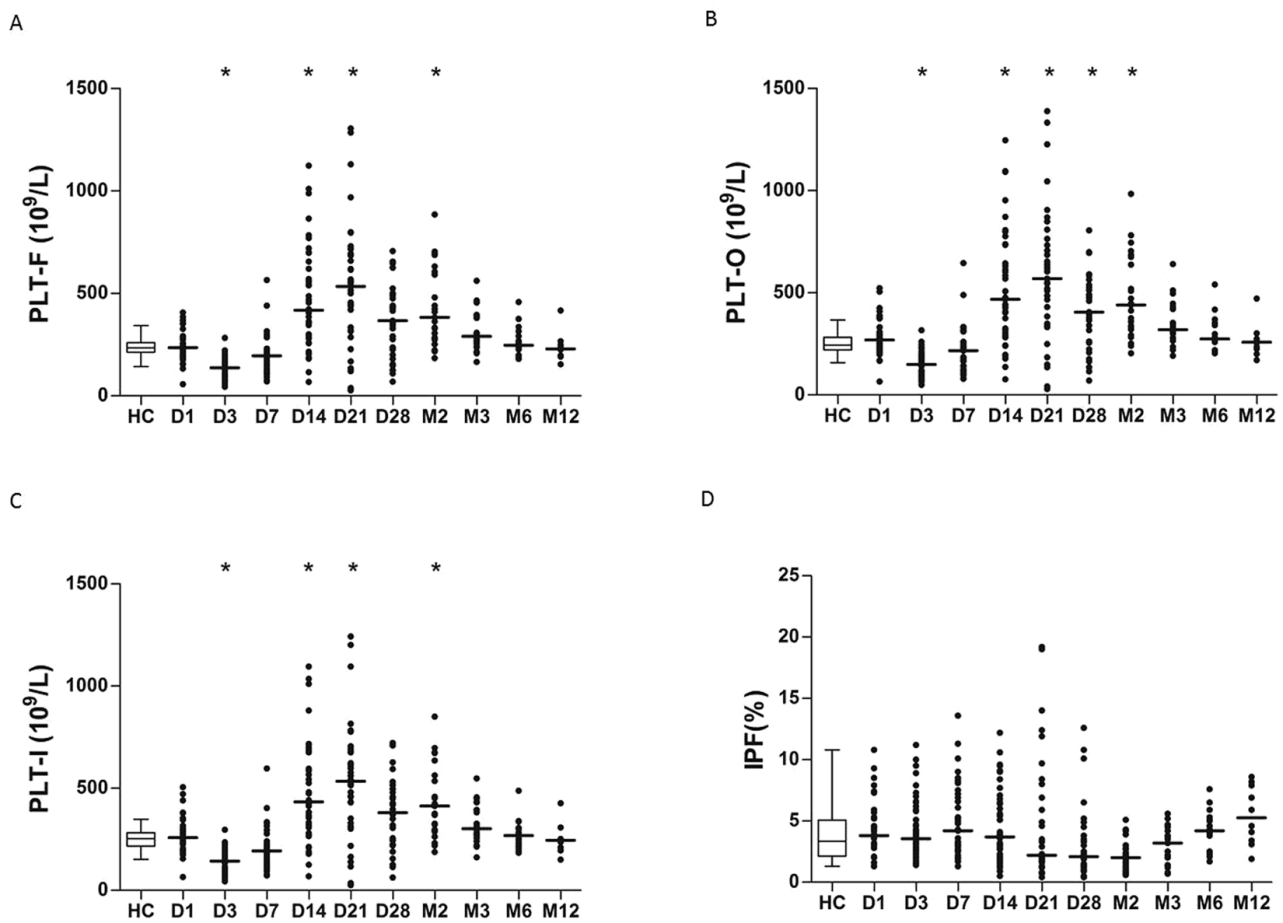
Thermal injury results in a dynamic platelet kinetic profile. (**A**) Platelet fluorescence count (PLT-F) across time (n = 39). (**B**) Platelet optical count (PLT-O) across time (n = 39). (**C**) Platelet impedance count (PLT-I) across time (n = 39). (**D**) Immature platelet fraction (IPF%) across time (n = 39). Differences in kinetics were compared to data from control cohort (n = 40) using a Mann-Whitney test; *p < 0.005.

The circulating number of platelets (PLT-I, O and F) was normal on the day of injury compared to control cohort. However, there was a significant reduction in all platelet counts on day 3 following thermal injury (Fig. 1A–C). PLT-F (r = −0.39), PLT-I (r = −0.37) and PLT-O (r = −0.38) nadir platelet counts correlated negatively with TBSA (p < 0.01). This nadir was followed by a rebound thrombocytosis in which platelet levels were significantly increased compared to healthy controls from day 14 to day 21 and again at month 2 post injury. The maximal rebound of platelet counts was apparent at day 21 (Fig. 1A–C). PLT-F (r = −0.56), PLT-I (r = −0.55) and PLT-O (r = −0.56) rebound platelet counts correlated negatively with TBSA (p < 0.01). IPF was not significantly elevated at any time points relative to control cohort (Fig. 1D) and failed to predict the platelet rebound.

### Platelet kinetics are different between septic and non-septic patients

**Figure 2:**
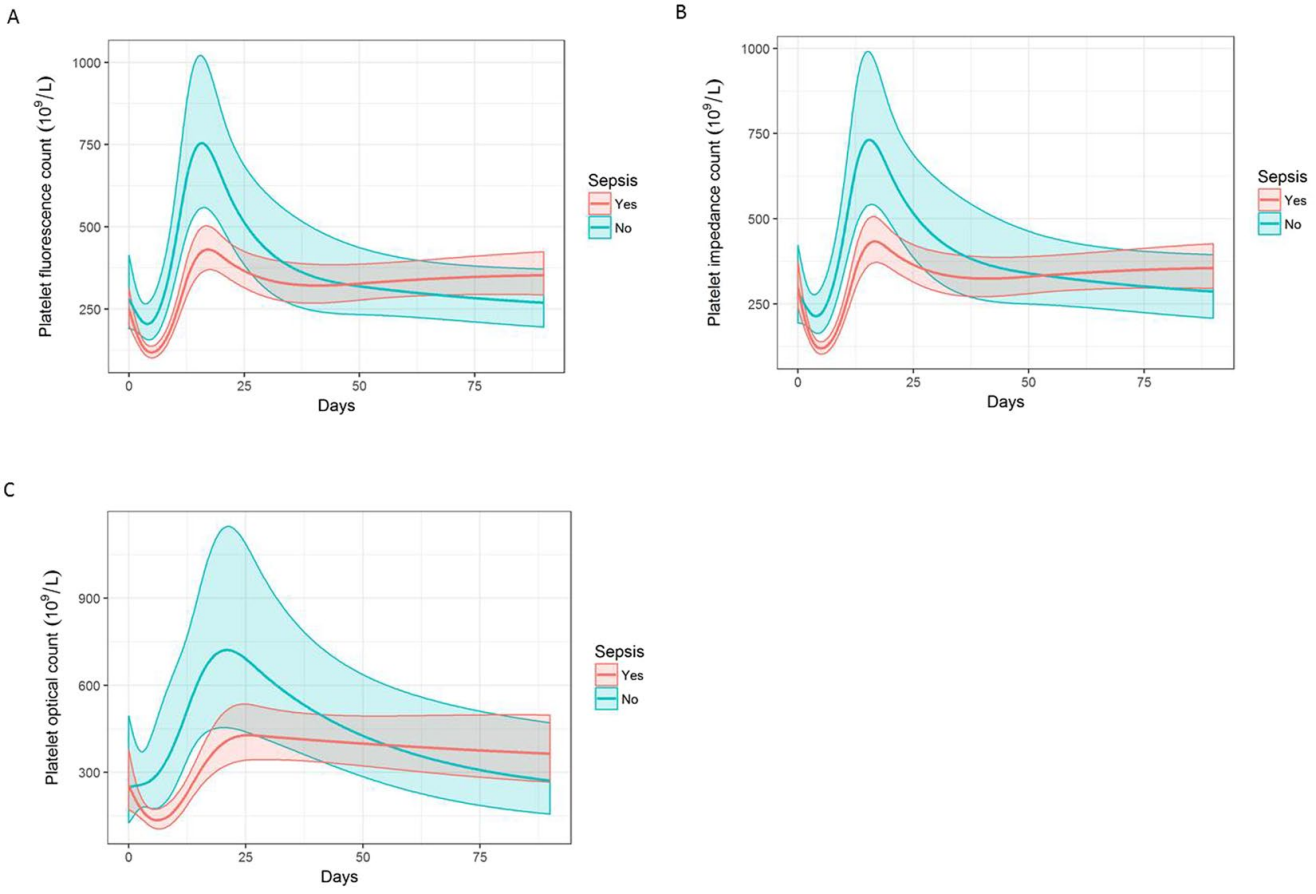
Platelet counts are different according to sepsis status. (**A**) Platelet fluorescence count (PLT-F) across time (n = 39). (**B**) Platelet impedance count (PLT-I) across time (n = 39). (**C**) Platelet optical count (PLT-O) across time (n = 39). Longitudinal analyses were performed using linear mixed-effects models to examine the relationship between time and platelet counts according to sepsis status (n = 39). Line represents predicted mean fixed effects; shaded area represents 95% confidence intervals.

Platelet levels were compared between patients who had 1 or more septic episodes and those who had no septic episodes. Septic patients had a significantly lower nadir platelet count at day 3 post injury as well as a significantly lower rebound platelet count at day 21 post injury (Fig. 2A–C). Having found a difference between the two patient cohorts, the discriminatory ability of these variables to distinguish between the septic and non-septic cohorts was assessed through the area under the receiver-operating characteristic curve (AUROC). The model performed best at day 3 post injury with an AUROC of 0.907 for PLT-F, 0.926 for PLT-I and 0.919 for PLT-O (Table 2). IPF was not significantly different between the septic and non-septic patient cohorts.

**Table 2:** Discriminatory power of platelet fluorescence count (PLT-F), platelet impedance count (PLT-I), platelet optical count (PLT-O), immature platelet fraction (IPF), white blood cell count (WBC), NEUT-Y and NEUT RI for sepsis at different time points.

Variable	Number of Patients	Number of Septic Patients	AUROC (95% CI)
**Day 1**			
PLT-F (10^9^/L)	27	10	0.669 (0.486, 0.851)
PLT-I (10^9^/L)	27	10	0.543 (0.345, 0.740)
PLT-O (10^9^/L)	27	10	0.583 (0.391, 0.776)
IPF (%)	27	10	0.530 (0.333, 0.727)
WBC (10^9^/L)	27	10	0.789 (0.645, 0.933)
NEUT-Y	27	10	0.770 (0.562, 0.979)
NEUT-RI	26	10	0.492 (0.264, 0.721)
**Day 3**			
PLT-F (10^9^/L)	30	9	0.907 (0.815, 1.000)
PLT-I (10^9^/L)	30	9	0.919 (0.834, 1.000)
PLT-O (10^9^/L)	30	9	0.926 (0.845, 1.000)
IPF (%)	30	9	0.507 (0.255, 0.760)
WBC (10^9^/L)	30	9	0.459 (0.248, 0.671)
NEUT-Y	30	9	0.733 (0.553, 0.913)
NEUT-RI	29	9	0.680 (0.483, 0.877)
**Day 7**			
PLT-F (10^9^/L)	24	8	0.880 (0.753, 1.000)
PLT-I (10^9^/L)	24	8	0.885 (0.760, 1.000)
PLT-O (10^9^/L)	24	8	0.875 (0.745, 1.000)
IPF (%)	24	8	0.768 (0.532, 1.000)
WBC (10^9^/L)	24	8	0.604 (0.379, 0.829)
NEUT-Y	24	8	0.779 (0.606, 0.951)
NEUT-RI	23	8	0.728 (0.534, 0.923)
**Day 14**			
PLT-F (10^9^/L)	26	8	0.774 (0.613, 0.935)
PLT-I (10^9^/L)	26	8	0.764 (0.601, 0.928)
PLT-O (10^9^/L)	26	8	0.787 (0.631, 0.943)
IPF (%)	26	8	0.774 (0.582, 0.966)
WBC (10^9^/L)	26	8	0.565 (0.351, 0.779)
NEUT-Y	25	8	0.850 (0.706, 0.994)
NEUT-RI	25	8	0.850 (0.674, 1.000)

Data was assessed through area under the receiver operating characteristic curve (AUROC) analysis and shown with 95% confidence intervals.

### Fragmented red cells (FRC) are generated following thermal injury

**Figure 3:**
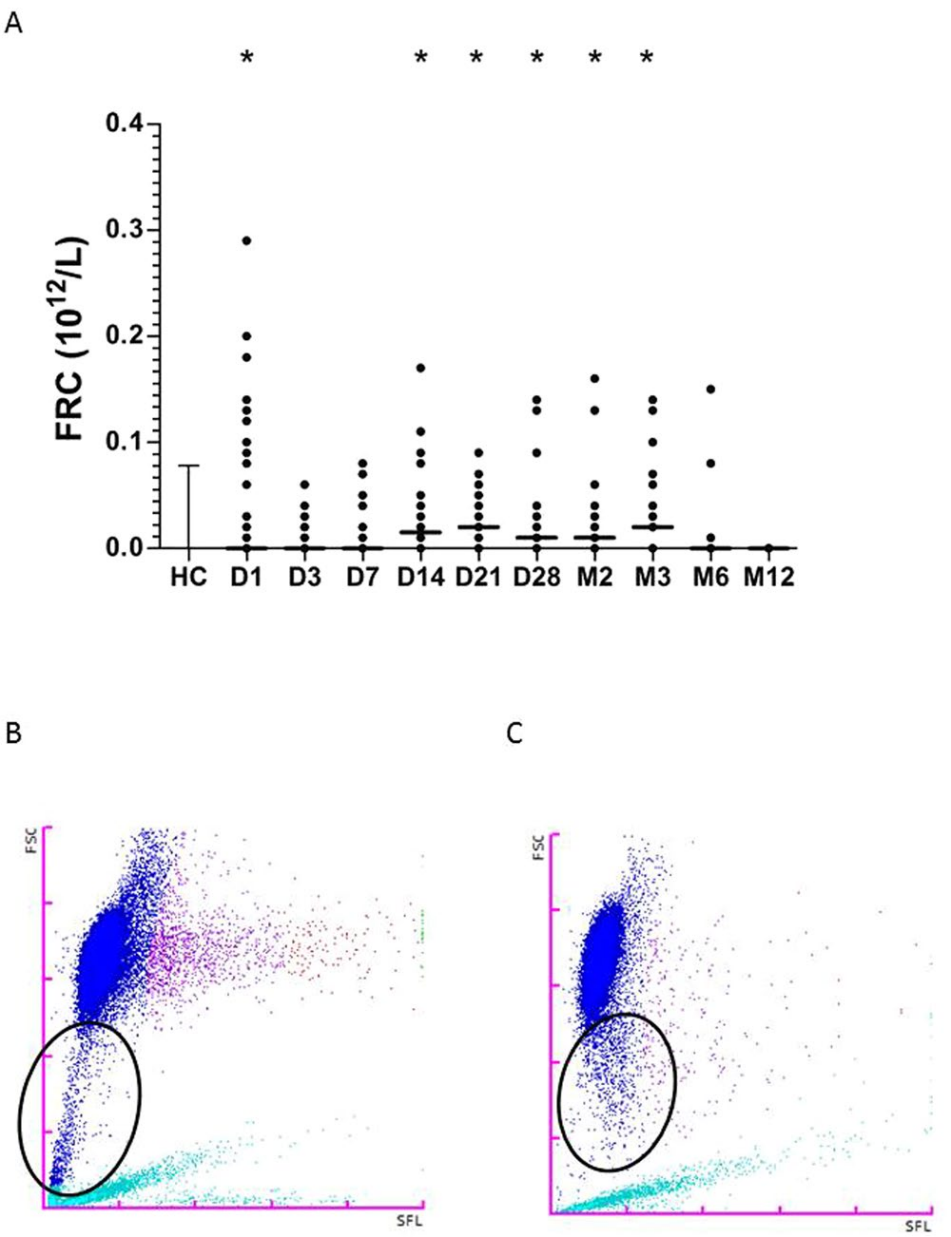
Thermal injury causes red cell lysis resulting in the production of fragmented red cells (FRC). (**A**) FRC across time (n = 39) differences in kinetics were compared to data from control cohort (n = 40) using a Mann-Whitney test; *p < 0.005. (**B**) A representative scatter graph displaying FRC (black circle) detectable on day 1 post injury. (**C**) A representative scatter graph displaying FRC (black circle) detectable on day 13 post injury. The intensity of the forward scatter (FSC, y-axis) indicates the cell volume and the side fluorescence indicates the amount of DNA and RNA present in the cell (SFL, x-axis). Colour key for scatter graphs; light blue = optical platelet count, dark blue = mature red blood cells, purple to orange = reticulocyte fractions.

Thermal injury resulted in an immediate significant increase in circulating FRC (Fig. 3A). Levels of FRC correlated positively with abbreviated burn severity index (ABSI) (p = 0.03, r = 0.37). FRC generated by thermal injury are small in size and ‘leak’ into the platelet gate causing a significant elevation in the PLT-O parameter compared to PLT-F (p < 0.005). FRC levels normalised on day 3 post injury relative to control cohort. Levels of FRC also became significantly elevated from day 14 to month 3 following thermal injury (p < 0.005, Fig. 3A). However, FRC generated at this time appeared much larger in size than at day 1 (Fig. 3B and C) and did not cause false elevation in platelet counts as they did not overlap into the platelet gates for PLT-I or PLT-O (Fig. 3C). Therefore, this second population appear to be a different phenotype of FRC. Importantly, the presence of FRC did not affect the discriminatory power of PLT-I or PLT-O as shown above.

### Thermal injury results in increased circulating white blood cells and neutrophils

**Figure 4:**
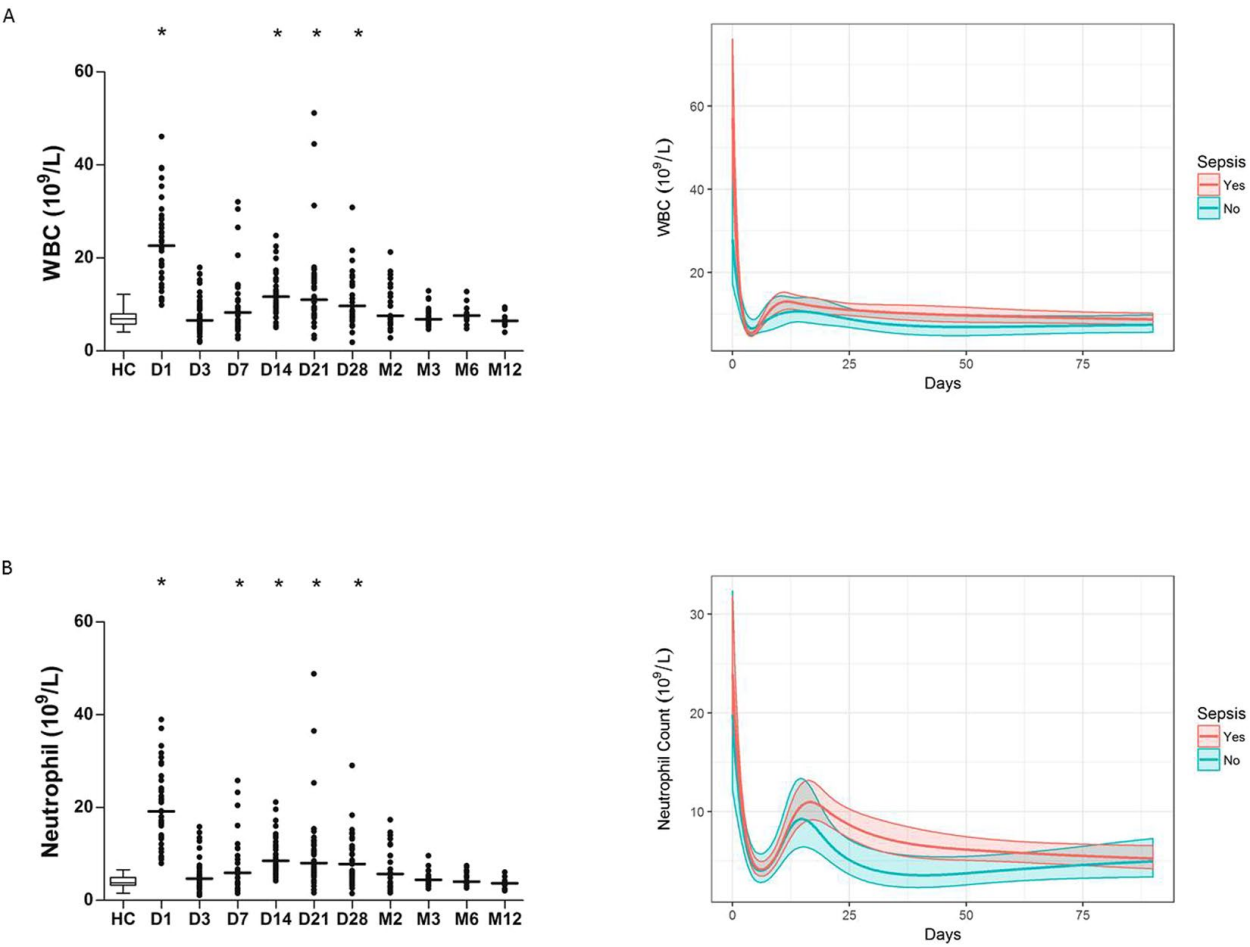
Circulating levels of white blood cells (WBC) and neutrophils are elevated post thermal injury. (**A**) White blood cell count (WBC) across time (n = 39). (**B**) neutrophil count across time (n = 39). Differences in kinetics were compared to data from control cohort (n = 40) using a Mann-Whitney test; *p < 0.005. Longitudinal analyses were performed using linear mixed-effects models to examine the relationship between time and WBCs or neutrophil count (n = 39). Line represents predicted mean fixed effects; shaded area represents 95% confidence intervals.

Thermal injury resulted in a significant elevation in the circulating levels of white blood cells (WBC) compared to control cohort. WBC were elevated on day 1 post-injury and again from day 14 to day 28 (Fig. 4A). Levels of WBC on day 1 post-injury correlated positively with percentage TBSA (p < 0.0005, r = 0.54). Levels of circulating neutrophils were also significantly elevated compared to the control cohort on day 1 post injury. Levels normalised on day 3 post injury relative to the control cohort and became significantly elevated from day 7 to month 2 following thermal injury (p < 0.005, Fig. 4B). Levels of circulating neutrophils on day 1 correlated positively with percentage TBSA (p < 0.005, r = 0.49). Neither WBC nor neutrophil counts alone provided any moderate/strong discriminatory ability for sepsis (Table 2).

### Thermal injury results in a change in extended neutrophil parameters

**Figure 5:**
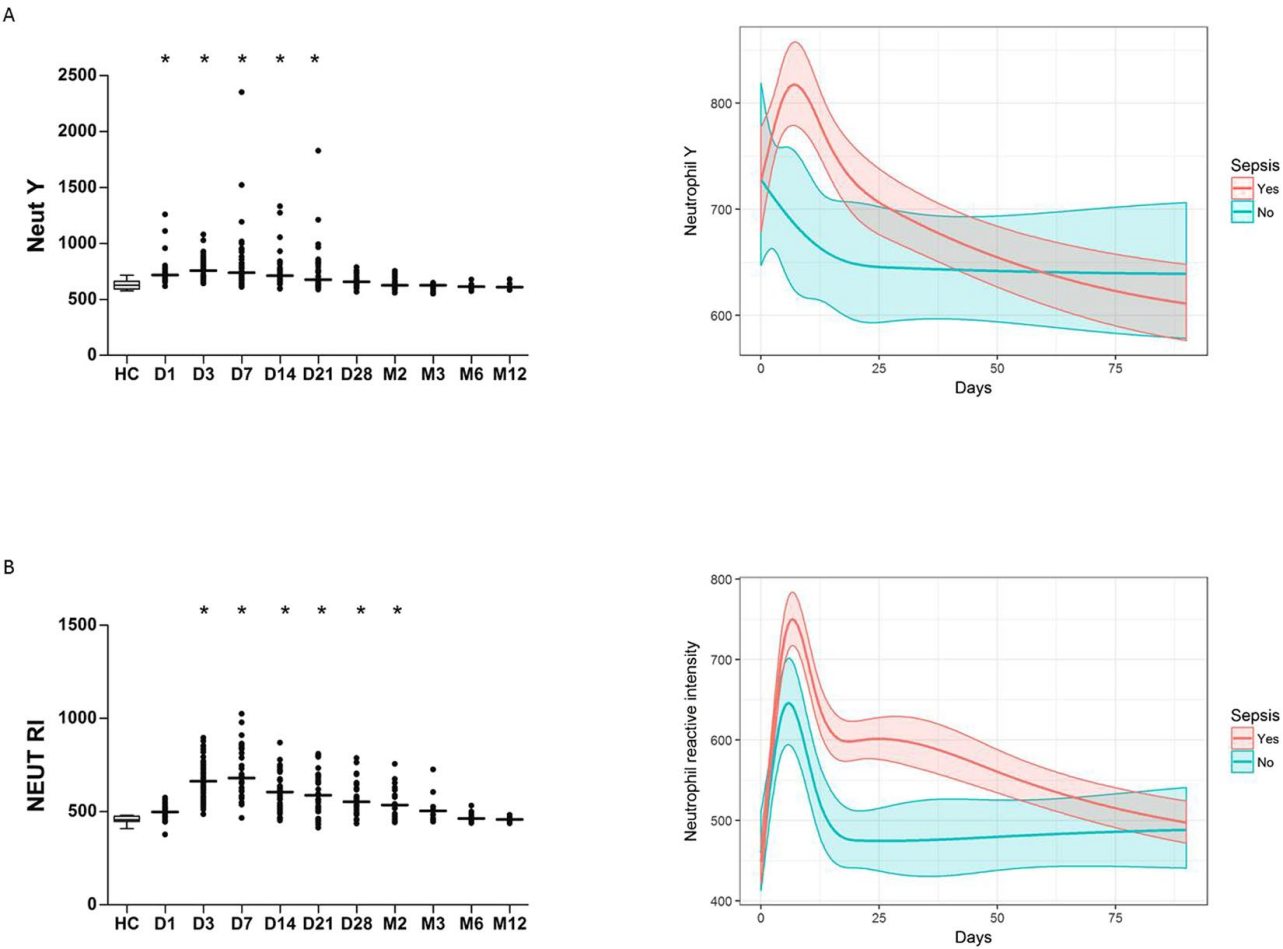
Burn injury results in a change of neutrophil phenotype and maturity. (**A**) Neut Y across time (n = 39). (**B**) neutrophil reactivity index (Neut RI) across time (n = 39). Differences in kinetics were compared to data from control cohort (n = 40) using a Mann-Whitney test; *p < 0.005. Longitudinal analyses were performed using linear mixed-effects models to examine the relationship between time and Neut Y or Neut RI count according to sepsis status (n = 39). Line represents predicted mean fixed effects; shaded area represents 95% confidence intervals.

NEUT-Y was significantly elevated on day 1 to day 21 post-injury (Fig. 5A). Septic patients had elevated NEUT-Y compared to non-septic patients from day 1 to day 21 post-injury (Fig. 5A). Having found a difference between the two patient cohorts, AUROC analysis was able to moderately differentiate between the two groups at day 1, 3, 7, 14 and 21 post-injury with AUROCs of 0.770, 0.733 0.779, 0.850 and 0.830 respectively.

Unlike NEUT-Y, neither NEUT-X nor NEUT-Z parameters were significantly different from the control cohort at any time point (data not shown). Only NEUT-GI remained normal for almost the entire time course, being significantly elevated only on day 3 post-injury compared to control cohort (data not shown). However, NEUT-GI had poor discriminatory power to differentiate between the septic and non-septic patients.

In contrast, NEUT-RI was significantly elevated from day 3 to month 2 post injury (Fig. 5B). Additionally, NEUT-RI was also significantly elevated in the septic patients from day 3 to day 28 post injury compared to the non-septic patients (Fig. 5B). Using AUROC analysis, NEUT-RI was able to moderately differentiate between the two cohorts at day 7 and 14 post-injury with AUROCs of 0.728 and 0.850 respectively (Table 2). The model had effectively no discriminatory power at day 1 following injury with AUROC of 0.492.

### Potential use of NEUT-Y and PLT-I as a biomarker of sepsis in burns

Having found a difference between platelet kinetics and extended neutrophil parameters between septic and non-septic patients we investigated the discriminatory power of a combination of the two. The two parameters chosen for this analysis were PLT-I and NEUT-Y as both show good to moderate discriminatory power alone and importantly both parameters are available on nearly all haematology analysers. The combined measurement using AUROC analysis of NEUT-Y and PLT-I gave good discriminatory power at day 3 (0.915), 7 (0.922) and day 14 (0.905) post injury (Table 3). However, the model had only moderate discriminatory power at day 1 (0.733) post-injury.

**Table 3:** Discriminatory power of a combination of NEUT-Y and platelet impedance count (PLT-I) for sepsis at different time points was assessed through area under the receiver operating characteristic curve (AUROC) analysis and shown with 95% confidence intervals.

Time Point	Number of Patients	Number of Septic Patients	Number of Non-Septic Patients	AUROC (95% CI)
Day 1	37	27	10	0.733 (0.524, 0.943)
Day 3	39	30	9	0.915 (0.827, 1.000)
Day 7	32	24	8	0.922 (0.823, 1.000)
Day 14	33	25	8	0.905 (0.793, 1.000)

Sepsis associated mortality is the leading cause of death following thermal injury and is of major clinical concern. We investigated if a combination of NEUT-Y and PLT-I could discriminate between septic patients who survived and did not survive. When the model was adjusted for mortality, AUROC analysis gave good discriminatory power at day 3 (0.974), 7 (0.969) and day 14 (0.910) post injury (Table 4).

**Table 4:** Discriminatory power of a combination of NEUT-Y and platelet impedance count (PLT-I) for sepsis adjusted for mortality at different time points was assessed through area under the receiver operating characteristic curve (AUROC) analysis and shown with 95% confidence intervals.

Time Point	Number of Patients	Number of Septic Patients	Number of Non-Septic Patients	AUROC (95% CI)
Day 1	37	27	10	N/A (N/A)
Day 3	36	27	9	0.974 (0.931, 1.000)
Day 7	32	24	8	0.969 (0.916, 1.000)
Day 14	33	25	8	0.910 (0.803, 1.000)

The model for Day 1 fails to converge due to a number of parameter combinations corresponding to zero observations in the data.

## Discussion

Diagnosis of sepsis remains a major challenge following thermal injury with many classically used criteria being masked by the ongoing SIRS^5^. Here, we report that a combination of routinely available cellular haematological parameters (PLT-I and NEUT-Y) shows good discriminatory power to predict later development of sepsis on day 3 post-injury (AUROC 0.915). The model showed improved performance when adjusted for mortality with an AUROC of 0.974.

Previous studies have shown that platelet kinetics follow a distinct pattern following thermal injury; with a nadir at day 3 and a rebound peak thrombocytosis at day 14 post injury^9^. The thrombocytopenic nadir could be caused by a number of mechanisms including; depression of bone marrow production, haemodilution, systemic platelet activation or consumption. Interestingly, measurement of the platelet activation marker soluble GPVI suggested that platelet activation was not significant in the acute phase of injury (Montague *et al*., personal communication). The observed rebound thrombocytosis was most likely a reactive response of the bone marrow to the consumption of platelets following thermal injury. However, here we report no elevation in IPF preceding the rebound thrombocytosis although it is possible, due to timing of our samples, that we may have missed the optimal peak in IPF in this cohort of patients. Therefore, a more comprehensive time course study with daily sampling is required to fully investigate platelet production and kinetics following thermal injury.

Importantly, several groups have reported that degree of the nadir of the platelet count is associated with sepsis^15, 16^. Here, we confirm that platelet nadir values are significantly different between septic and non-septic patients. Additionally, in agreement with previous literature, the platelet nadir alone has moderate discriminatory power for predicting later development of sepsis in our cohort (PLT-I = 0.919, PLT-O = 0.926 and PLT-F = 0.907). Importantly, all three methods of platelet count have comparable discriminatory power suggesting that interference by FRC in the PLT-I and PLT-O counts at day 1 was not a significant issue. Additionally, haematocrit (HCT) levels within 24 hours of injury remain comparable to levels seen in healthy individuals. This is followed by a significant decrease from day 3 to month 2 post injury which is indicative of potential haemodilution (Supplementary Figure 1) and may also contribute to the fall in platelet counts at day 3. However, platelet levels rebound at day 21 post injury when HCT levels are still significantly reduced. Importantly, all patients received a standardised burn resuscitation protocols as per Parkland Formula and as such have received equivalent fluid resuscitation (average = 5.4 mls/kg/%TBSA, standard deviation = 2.1).

It is important to note that in patients with severe thermal injuries platelet counts can be falsely elevated^17, 18^. There are a number of sources which can induce error into the traditional methods of platelet counting; namely impedance or optical counting^11, 19^. Thermal injury results in the direct destruction of red blood cells within skin capillaries leading to the appearance of FRC, commonly referred to as microspherocytes^20^. FRC are products of erythrocyte lysis present in a number of pathological conditions but almost completely absent in healthy individuals^21, 22^. Due to the nature and variation in size of FRC quantification has been extremely difficult and has traditionally relied upon visual examination of blood films^23^. Here we utilise a rapid and novel method which allows accurate FRC quantification for the first time in thermal injury using automated fluorescence flow cytometry. Indeed, burn injured patients had significantly higher levels of FRC on day 1 of injury compared to healthy controls but these completely disappear by day 3. ABSI significantly correlated with levels of FRC measured within 24 hours of injury. This elevation in FRC was also accompanied by a false elevation in the PLT-I and PLT-O counts. However, the PLT-F count remained totally unaffected by FRC.

Interestingly, a second population of FRC also becomes detectable at later time points following thermal injury which are larger in size (Fig. 3C). One likely source of this second population is the occurrence of disseminated intravascular coagulation (DIC)^21, 24^. DIC is characterised by the formation of microthrombi in the microvasculature and is commonly associated with sepsis, multi-organ failure and infection. Formed thrombi can cause the fragmentation of red cells which may potentially explain the appearance of a larger size FRC, also known as schistocytes. Our data suggest that the XN-1000 may also be able to differentiate between smaller spherocytes and schistocytes with application of appropriate gating between the large and smaller populations of FRC. Interestingly, it has been previously shown that products of red cell lysis may induce defence actions of neutrophils (NETosis) during sepsis^24, 25^. Therefore, this population may also play a functional role in combating infections following thermal injury.

Thermal injury also results in a marked neutrophil dysfunction which is associated with sepsis^6, 26–28^. One potential contributor to reduced neutrophil function is the IG population which are released prematurely from the bone marrow in response to stress. Released immature neutrophils are classically banded in morphology and exhibit defective neutrophil function. Importantly, quantification of circulating IG has shown to be able to discriminate between septic and non-septic patients with a sensitivity of 89.2% and a specificity of 79.4%^12^. However, in this study we performed a more comprehensive analysis of neutrophil phenotype and maturity by investigating five additional novel parameters; NEUT-Y, NEUT-X, NEUT-Z, NEUT-GI and NEUT-RI. All five provide more detailed information of the properties of the entire neutrophil population. In this study only NEUT-Y and NEUT-RI demonstrated any significant changes in thermal injury patients compared to control cohort. Septic patients gave significantly higher NEUT-Y and NEUT-RI values compared to non-septic patients and control cohort. This is in contrast to a recent study by Luo *et al*. who demonstrated that NEUT-X, NEUT-Y and NEUT-Z can all be used for diagnosis of sepsis in patients with tumours^29^.

The elevation in NEUT-Y and NEUT-RI observed in our cohort is probably mediated by the release of immature neutrophils which have classical banded nuclear morphology (e.g. IG, promyelocytes, myelocytes or metamyelocytes). This subset of cells is potentially an additional phenotype of immature neutrophils, e.g. myelocyte or metamyelocyte, which will contribute to the increased NEUT-Y and NEUT-RI values. Using similar methodology, Stiel *et al*. have reported an increase in nuclear acid content in septic patients with DIC. Additionally, in this study the level of neutrophil nucleic acids had strong discriminatory power of diagnosis with a sensitivity of 90.91% and a specificity of 80.60%^30^. In our study we expanded upon this by investigating both the early predictive power and later diagnostic potential of extended neutrophil parameters. Of note, in 222 patients Cornet *et al*. reported that NEUT-Y and NEUT-RI could be used to predict an increase in IG in septic or non-infectious/reactive patients. Interestingly, extended neutrophil parameters distinguished between infectious and non-infectious elevations in IG count. NEUT-RI and NEUT-Y therefore have potential to improve the interpretation of elevated IG levels in patients and providing added value to clinical practice^31^. Therefore, measurement of the total neutrophil population and any morphological changes may be more informative and aid in early identification of septic patients compared to quantifying IG count alone.

Previously it has been shown that a combination of laboratory or clinical markers provide better discriminatory power for sepsis^10^. As we report a difference in platelet counts and neutrophil phenotype between septic and non-septic patients the discriminatory power of PLT-I and NEUT-Y was also assessed in combination. PLT-I was chosen in this statistical model over PLT-F and PLT-O as it is a routine clinical measurement available on nearly all commonly used haematological analysers. Importantly at all time points, PLT-I, PLT-O and PLT-F had comparable discriminatory power for predicting the later development of sepsis. In this study, a combination of PLT-I and NEUT-Y had a comparable discriminatory power relative to that of a single parameter of PLT-I. The model had AUROC of 0.733, 0.915, 0.922 and 0.905 at days 1, 3, 7 and 14 respectively. Of note, the median time to first episode of multiple organ failure was 4 days. Therefore our model at day 3 is not influenced by complications e.g. liver failure or cardiac dysfunction.

Sepsis associated mortality remains significantly high post thermal injury. As PLT-I alone has poor discriminatory power for mortality we further investigated if a combination of PLT-I and NEUT-Y could discriminate between septic patients who did and did not survive their injuries. The model showed improved performance with an AUROC of 0.974 (0.931, 1.000) on day 3 post injury. However, with 27 sepsis cases observed in 39 patients there is a risk of model overfitting and the AUROC should therefore be interpreted with caution. These findings highlight the potential utility of a combination of biomarkers for the early prediction and/or diagnosis of sepsis and its associated mortality.

A limitation of the current study is the limited sample size and lack of formal power calculation as this study was designed to be exploratory and hypothesis generating in nature and not confirmatory. Therefore a much larger study is required to confirm these novel findings and evaluate this novel clinical model.

In conclusion, a combination of routinely available biomarkers (PLT-I and NEUT-Y) show potential for the early diagnosis of sepsis and associated mortality post-burn injury. Given the rapidity (~40 seconds) and robustness of this extended full blood count panel coupled with the availability of point of care (POC) versions this technology therefore has excellent potential to translate into routine clinical use to improve care of critically ill patients.

## Methods

### Study group

Thirty nine consecutive patients admitted to the Tertiary referral Burns Centre with a burn size of ≥15% total body surface area (TBSA) were recruited into a prospective cohort study within 24 hours of their injury (aged between 16–99 years). Patients were excluded from the study if; patient had multiple injuries with an injury severity score >25, injury was chemical or deep electrical, decision not to treat due to severity of injury or premorbid conditions (including congestive heart failure, malignancy, multiple limb amputations or patients receiving glucocorticoid treatment). Blood samples from patients and healthy volunteers were collected into standard BD Vacutainers® (Becton Dickinson, Oxford, UK) containing ethylenediaminetetraacetic acid (EDTA). Blood samples were collected at intervals following injury (day 1 [<24 hours post-injury], day 3 [+/−1 day], day 7 [+/−1 day], day 14 [+/−3 days], day 21 [+/−3 days], day 28 [+/−3 days], month 2 [+/−3 days], month 3 [+/−7 days], month 6 [+/−7 days] and month 12 [+/−7 days]). A diagnosis of sepsis was made when at least three of the six consensus criteria agreed in 2007 by the American Burn Association (ABA) were met along with either (1) a positive bacterial culture or (2) evidence of a clinical response to antibiotics was detected^32^.

### Analysis of blood cell distributions

Whole blood cell counts were performed on EDTA anticoagulated blood using the Sysmex XN-1000 haematology analyser (Sysmex UK, Milton Keynes, UK). The analyser utilises three primary analysis principles; fluorescence flow cytometry, direct current (DC) detection with hydrodynamic focussing and SLS haemoglobin detection. There are 5 standard channels of analysis. The WNR channel evaluates white blood cell count (WBC), basophils and nucleated red blood cells. The WDF channel provides a count of the neutrophils, lymphocytes, eosinophils, monocytes and immature granulocytes (IG). Extended neutrophil parameters are also available in this channel e.g. NEUT X, Y, Z. NEUT GI and NEUT RI were obtained after further analysis of the data by Sysmex UK. The WPC channel evaluates white precursor cells with high sensitivity and specificity. The RET channel provides a count of reticulocytes in addition to measurements of red blood cell formation and maturity. This channel is also used to determine the platelet optical count (PLT-O). The PLT-F channel is a specialised fluorescence-optical analysis exclusively for platelets. The PLT-F parameter utilises traditional fluorescence flow cytometry in which platelets are stained with oxazine; an RNA binding dye which eliminates any interference mediated by cellular debris. A measurement of platelet production (Immature Platelet Fraction) is also in this channel.

Quality control material (XN check) was tested on a daily basis to ensure instrument performance throughout the study. The instrument was also enrolled into a national external quality assurance scheme (UKNEQAS, Watford, UK). To determine normal ranges for each cellular parameter, blood samples from 40 healthy volunteers (control cohort) were analysed and normal ranges are represented as the mean value +/−2 standard deviations.

### Statistical Analysis

Data were checked for normality using the Shapiro-Wilk test. Continuous variables were then compared using a Mann-Whitney test with a Bonferroni correction for multiple comparisons, an unpaired t-test or a 2-way ANOVA where appropriate. Categorical variables were compared using a Chi-squared test. Logistic regression analyses were conducted to examine the relationships between cellular kinetics at pre-specified sample times (e.g. day 7) and the presence of sepsis. Discriminatory power was assessed through the area under the receiver operator characteristic curve (AUROC). Longitudinal analyses were performed using linear mixed-effects models. Sample day was included in these models as a restricted cubic spline to allow for a flexible non-linear relationship between time and the response variable. Analysis was performed using the statistical software packages SPSS (IBM) and R version 3.0.1 (http://www.r-project.org) together with the Ime4, effects, rms and pROC packages.

### Study approval

Ethical approval for the study was granted by a UK NHS research ethics committee (Reference 12/EM/0432). Where possible, written informed consent was received from participants prior to their inclusion in the study. Due to the severe nature of the injuries being studied, the ethics committee approved the use of a legal consultee, either personal or nominated, if the patient was not initially able to consent for inclusion in the study themselves. When the patient was able, they were approached to give written consent to continue to participate in the study.

## Electronic supplementary material

Supplementary figure 1
