# Supplementary material for: Changes in novel haematological parameters following thermal injury: A prospective observational cohort study
Source: Sci Rep. 2017 Jun 12;7:3211. doi: 10.1038/s41598-017-03222-w (PMC5468303; doi:10.1038/s41598-017-03222-w)
Supplement: Supplementary file 1 — Supplementary figure 1 [file 41598_2017_3222_MOESM1_ESM.pdf]

Changes in novel haematological parameters following thermal injury

A prospective observational cohort study

*Dinsdale RJ[1,2], Devi A[1,2], Hampson P[1,2], Wearn CM[1,2,5], Bamford AL[1,5], Hazeldine J[2,4], Bishop J[4], Ahmed S[3], Watson C[3], Lord JM[1,2,4], Moiemen N[1,5], Harrison P[1,2].

[1]Healing Foundation Birmingham Centre for Burns Research, Birmingham UK.

[2]Institute of Inflammation and Ageing, University of Birmingham, Birmingham UK

[3]Department of Haematology, Queen Elizabeth Hospital, Birmingham, UK.

[4]NIHR Surgical Reconstruction and Microbiology Research Centre, University Hospitals Birmingham NHS Foundation Trust, UK.

[5]Queen Elizabeth Hospital Birmingham, University Hospitals Birmingham NHS Foundation Trust, Mindelsohn Way, Birmingham, B15 2WB, UK.

Corresponding Author: Robert Dinsdale, Institute of Inflammation and Ageing, University of Birmingham, Birmingham, B15 2TT UK. Tel: (+44)121 371 3264. Email: RXD325@bham.ac.uk

Fax: (+44)121 374 3203.

For reprints: Robert Dinsdale, Institute of Inflammation and Ageing, University of Birmingham, Birmingham B15 2TT UK. Tel: (+44)121 371 3264. Email: RXD325@bham.ac.uk

Fax: (+44)121 374 3203.

This work was funded by The Healing Foundation and National Institute for Health Research. The Sysmex XN-1000 Analyser was funded by the Queen Elizabeth Hospital Charity.

Short title: Novel Haematological Parameters Following Thermal Injury

Supplementary Figures

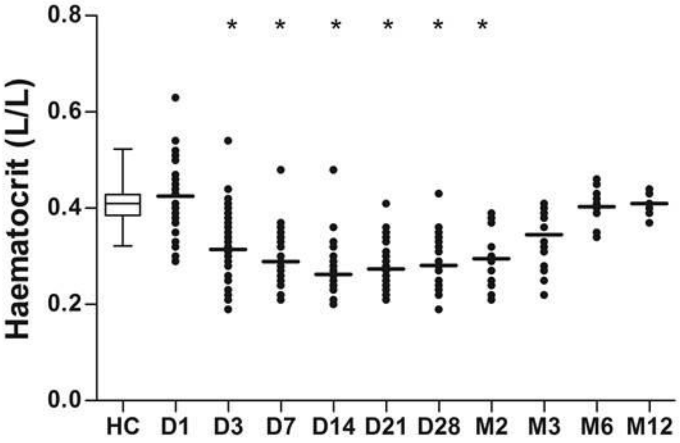

Supplementary figure 1. Thermal injury results in a reduction in haematocrit levels from day 3 to month 2 post injury. A, Haematocrit levels (HCT) across time (n=39). Differences in kinetics were compared to data from control cohort (n=40) using a Mann-Whitney test; *p<0.005.
